# Effect of pre-slaughter fasting time on carcass yield, blood parameters and meat quality in broilers

**DOI:** 10.5713/ab.23.0262

**Published:** 2023-11-01

**Authors:** Xuezhuang Wu, Yahao Zhou, Zhentao Lu, Yunting Zhang, Tietao Zhang

**Affiliations:** 1 College of Animal Science, Anhui Science and Technology University, Fengyang, 233100, China; 2 Institute of Special Animal and Plant Sciences, Chinese Academy of Agricultural Sciences, Changchun 130112, China

**Keywords:** Broilers, Carcass Yield, Fasting, Glucose Metabolism, Meat Quality

## Abstract

**Objective:**

The aim of this study was to evaluate the effect of pre-slaughter fasting time on carcass yield, meat quality, blood parameters and glucose metabolism in broilers.

**Methods:**

Four hundred and fifty Arbor Acres (AA) broilers at 42 days of age were divided into 5 groups with 6 replicates in each group and 15 chickens as one replicate. Following this period, broilers from each group were distributed among five groups according to pre-slaughter fasting period as 4, 8, 12, 16, or 20 h.

**Results:**

With increasing fasting time, the carcass yield (p<0.01), the breast muscles yield (p<0.01) and the thigh yield (p<0.01) of the broilers were all linearly increased. With increasing fasting time, the L* values (p<0.01), cooking loss (p = 0.020), moisture content (p<0.01) in the leg muscles linearly downregulated, while the drip loss (p = 0.043), pH_45 min_ (p<0.01) and pH_24 h_ (p<0.01) were linearly upregulated. A trend for a lower (p = 0.071) shear force in the leg muscles was also observed in broilers fasted for longer time. Similar results were also found in breast muscles. The different fasting treatments did not influence the breast muscles glycogen content (p>0.10), while the increase of fasting time resulted in a linear decrease of the blood glucose (p = 0.021) and, more specifically, the glycogen content of the liver and leg muscles (p<0.001). With increasing fasting time, the aspartate transaminase (p<0.01), uric acid (p<0.01), and triglycerides (p<0.01) in serum linearly downregulated, while the alanine aminotransferase was linearly upregulated.

**Conclusion:**

The results of this study show a significant influence of fasting time on carcass yield and meat quality in broilers. Moderate fasting (8 to 12 h) before slaughter can reduce the weight loss of broilers. Prolonged fasting (≥16 h) increased body weight loss, decreased slaughtering performance and fluctuating blood indexes of broilers.

## INTRODUCTION

Before slaughter, broilers were stimulated by factors such as fasting, capture, cage loading and transportation, which seriously affected the welfare of broilers and had a negative impact on meat quality [1,2]. Fasting time is one of the key factors affecting meat quality and slaughter rate [3–5]. It is common practice to fast broilers for several hours before slaughter [6–8]. Fasting can empty the chyme in the digestive tract of animals, reduce the possibility of carcass contamination by feces during slaughter, and also reduce transportation stress and mortality [9–11]. Too short or unrestrained fasting time will lead to waste of feed taken several hours before slaughter and increase transport stress [12]. However, excessive fasting will increase the weight loss of broilers [13], reduce the glycogen content [14], and then reduce the slaughter weight [15]. A reasonable fasting time will ensure optimal weight loss, feed waste, and animal stress for broilers.

After fasting, with the nutritional supply of broilers stopped, the metabolic activity changes from anabolism to catabolism, from fat production to fat decomposition, and gluconeogenesis is strengthened [16]. This will lead to increased weight loss [17], resulting in economic losses for the slaughterhouse. In addition, serum biochemical parameters in broilers will change after fasting [13]. After slaughter, the oxygen supply of body is stopped while energy is still needed for cell activities under anaerobic conditions, which will lead to glycolysis and lactic acid accumulation, thus reducing the pH value of muscles and affecting the color and water retention of meat [16–18]. Excessive fasting before slaughter will lead to a large consumption of muscle glycogen while the bird is alive leading to less glycogen content in muscles after slaughter, and less lactic acid produced by glycolysis.

Arbor Acres (AA) broilers are characterized by fast growth rate, high adaptability, high feed conversion rate and good carcass quality. They were introduced into China in the early 1980s, and AA broilers are now a common commercial broiler breed and widely farmed in the broiler industry. At present, there are few studies on the effects of fasting time on meat quality and glucose metabolism in broilers. Therefore, this experiment took AA broilers as the research object to explore the effects of fasting time before slaughter on body weight loss, meat quality, glucose metabolism and serum biochemical indexes, to provide theoretical reference for actual production.

## MATERIALS AND METHODS

The animal protocol for this experiment was approved by the Animal Care Committee of the Anhui Science and Technology University (No. 2023007). Animals were maintained and processed in accordance with the Anhui Science and Technology University Guide for the Care and Use of Laboratory Animals.

### Animals and treatments

Four hundred and fifty AA broilers (2,612.15±65.01 g, from Animal Husbandry Science Park of Anhui University of Science and Technology) at 42 days of age were divided into 5 groups with 6 replicates in each group and 15 chickens as one replicate. At 7:00 am on d 42, all experimental broilers were weighed (initial body weight, IBW), fasted and provided with drinking water. The broilers of fasting 4 h group (F4, transportation for 2 h, and laboratory rest for 2 h), 8 h group (F8, fasting for 4 h, transportation for 2 h, and laboratory rest for 2 h), 12 h group (F12, fasting for 8 h, transportation for 2 h, and laboratory rest for 2 h), 16 h group (F16, fasting for 12 h, transportation for 2 h, and laboratory rest for 2 h), and 20 h group (F20, fasting for 16 h, transportation for 2 h, and laboratory rest for 2 h) were transported to the laboratory at 7:00 am, 11:00 am, 3:00 pm, 7:00 pm and 11:00 pm. All birds were weighed as final body weight (FBW) before slaughter and killed by cervical dislocation.

Weight losses (WL) were calculated according to the following formula:


WL=(IBW-FBW)/IBW×100%

### Slaughter performance

Broilers were slaughtered according to the factory production line process, with an ambient temperature of 20°C±2°C. Carcasses were weighed and deboned 1 d after slaughter. After slaughter, all feathers on the skin were removed by a de-feathering and washing machine. The paws were removed, followed by evisceration, and the weight determined again to obtain the carcass weight. The weight of the breast and thigh muscles is obtained by separating them with blunt scalpels and scissors and removing fat with tweezers. Abdominal fat was separated, weighed, and abdominal fat yield was calculated. All variables were expressed in absolute weight and as percentages of the carcass weight.

### Blood parameters

Blood samples were collected from the wing vein of 60 broilers (2 broilers/treatments/replicates). Then the blood samples were centrifuged at 3,000 *g* for 15 min. Serum was separated and stored at −80°C for further analyses. The concentrations of serum total protein, albumin, uric acid (UA), triglycerides, total cholesterol, high-density lipoprotein cholesterol, low-density lipoprotein cholesterol cholesterol were analyzed by an automatic biochemistry analyzer (Hitachi 7020; Hitachi High Technologies, Inc., Ibaraki, Japan). The assay kits for the above were supplied by Zhongsheng Beikong Biotechnology LLC, Beijing, P. R. China.

### Meat quality

After slaughter, about 8 g of the liver and meat samples were placed in cryo-storage tubes and immersed in liquid nitrogen for the determination of glycogen and other indicators. The rest was placed in a 4°C refrigerator for measuring muscles pH value, meat color, cooking loss, drip loss, and shear force.

The pH value was measured 45 min after slaughter (pH_45 min_) and 24 h (pH_24 h_) after slaughter with a Model pH/ISE portable pH meter. Meat color was measured 24 h postmortem using a CR400 Minolta Chroma meter (Osaka, Japan). Each sample was measured 3 times and its average value were taken for statistics. The drip loss percentage was determined during 24 h of storing the meat samples (weighing approximately 100 g) according to Xiong et al [19]. During that time, the samples were placed in plastic bags and kept at 4°C for 24 h. The cooking loss of meat was determined based on weight method according to Przybylski et al [20]. The shear force was measured by the People’s Republic of China Agricultural Industry Standard NY/T 1180-2006 ‘Determination of Meat Tenderness-Shearing Force Determination Method’. The meat samples were taken and heated in a constant temperature water bath with a power of 1,500 W at 80°C. When the center temperature of the meat sample reached 70°C, it was taken out and cooled to a center temperature of 4°C. A circular sampler with a diameter of 1.27 cm was used to drill and cut the meat samples along the direction parallel to the muscle fibers, and the length of the hole samples was not less than 2.5 cm. The final value used for analysis was the average of 6 shear force measurements. Accurately weigh about 10 g of meat sample, dry it to constant weight at 105°C, and calculate the muscles moisture content.

### Glycometabolism

Liver and muscles glycogen were determined by colorimetric method, and the operation was carried out in strict accordance with the instructions in the kit. Liver/muscles glycogen determination Kit were purchased from Nanjing Jiancheng Institute of Biological Engineering.

### Statistical analysis

Statistical Analysis System version 9.2 (SAS Institute, 2009; Cary, NC, USA) [21] was used to analyze the data. Duncan’s new multiple range test were used to analyze the difference between any two groups. The linear contrasts of one-way analysis of variance were further used to determine the effects of the different Fasting time (4, 8, 12, 16, and 20 h). Statistical significance was set at p<0.05.

## RESULTS

### Body weight loss and carcass quality traits

Table 1 shows the effects of fasting time on the carcass quality traits and body weight loss in broilers. In this study, there was no significant difference in the IBW of broilers between groups (p>0.10; Table 3), while the increase of fasting time resulted in a linear decrease of the slaughter body weight (p = 0.004) and, and body weight loss and loss rate before slaughter increased with increasing length of fasting time (p<0.05). With increasing fasting time, the carcass yield (p<0.01), the breast muscles yield (p<0.01) and the thigh yield (p<0.01) of the broilers were all linearly increased.

### Muscle quality

Table 2 and Table 3 show the effects of fasting time on muscle quality in broilers. With increasing fasting time, the L* values (p<0.01), cooking loss (p = 0.020), moisture content (p<0.01) in the leg muscles linearly downregulated, while the drip loss (p = 0.043), pH_45 min_ (p<0.01) and pH_24 h_ (p<0.01) were linearly upregulated. A trend for a lower (p = 0.071) shear force in the leg muscles was also observed in broilers fasted for longer time. Similar results were also found in breast muscles. With increasing fasting time, the L* values (p<0.01), cooking loss (p<0.01), moisture content (p<0.01) in breast muscles linearly downregulated, while the pH_24 h_ (p<0.01) was linearly upregulated.

### Glucose metabolism

Table 4 shows the effects of fasting time on glucose metabolism in broilers. The different fasting treatments did not influence the breast muscles glycogen content (p>0.10), while the increase of fasting time resulted in a linear decrease of the blood glucose (p = 0.021) and, more specifically, the glycogen content of the liver and leg muscles (p<0.001).

### Serum biochemical indexes

Table 5 shows the effects of fasting time on serum biochemical indexes in broilers. With increasing fasting time, the aspartate transaminase (AST) (p<0.01), UA (p<0.01), and triglycerides (TG) (p<0.01) in serum linearly downregulated, while the alanine aminotransferase (ALT) was linearly upregulated. The serum TG contents in the F4 of the broilers were significantly higher than those of the F16 and F20 (p<0.05).

## DISCUSSION

To improve meat quality and reduce fecal and microbial contamination, it is common practice to withhold food from broiler chickens for several hours before slaughter.

In this study, body weight loss of broiler chickens increases with the prolongation of fasting time, and broiler chickens experience the greatest relative weight loss after 20 hours of fasting. The weight loss is 0.40% per hour when fasting for 0 to 4 hours, 0.34% per hour when fasting for 0 to 8 hours, 0.28% per hour when fasting for 0 to 12 hours, and 0.25% per hour when fasting for 0 to 16 hours, and 0.22% per hour when fasting for 0 to 20 hours. Fasting causes body weight loss mainly due to three reasons: the excretion of intestinal contents, the loss of muscles’ water, and the oxidation metabolism of nutrients for energy supply [1,22]. Fasting for 6 to 10 h resulted in reducing the likelihood of carcass of broilers, mainly due to the excretion of intestinal contents [11]. The body weight loss is slightly slower from 12 to 20 hours of fasting, mainly due to metabolic activity and water loss. This study confirms that muscle moisture content also decreases with increasing fasting time. Fasting before slaughter causes economic losses to the slaughterhouse due to weight loss.

Ali et al [16] found that fasting for 24 hours did not affect the fat content, abdominal fat rate and fatty acid composition of chicken. However, the reduction of water content in chicken also affects the final carcass yield and meat quality, such as meat color and tenderness [9,23]. Most of the water in the animal body is in the muscles, accounting for 69% to 75% of the total muscle weight [24]. The loss of water in the process of slaughtering will inevitably cause economic losses in the slaughterhouse. This study showed that the water content of the chest muscles and leg muscles decreased with the increase of fasting time, but the water content of the chest muscles and leg muscles was significantly lower than the control group only after 24 hours of fasting.

Blood lipids mainly consist of fatty acids and cholesterol, and their concentrations depend on the absorption and excretion in the intestines, as well as absorption and secretion in cells [25]. Normally, the concentration of fatty acids in the blood will temporarily increase after ingestion [26]. The animal’s intestines absorb free fatty acids, which are synthesized into triglycerides in the liver and then transported into the blood in the form of lipoproteins [27]. Triglycerides in the blood are kept in dynamic equilibrium under normal conditions [17]. Fasting inevitably leads to the oxidative metabolism of fatty acids to supply energy. The rate of triglyceride synthesis in the liver slows down and the oxidative decomposition for energy supply accelerates after fasting, thus causing a decrease in the content of plasma triglycerides. Research results show that fasting before slaughter will reduce serum triglyceride levels in broiler chickens [28,29]. This study indicates that the serum triglyceride content in broiler chickens decreases with the prolongation of fasting time, and during fasting, triglycerides in the blood of broiler chickens are converted into fatty acids for energy supply, but triglycerides in the tissues have not yet been converted into fatty acids during short-term fasting.

Fasting before slaughter will lead to the depletion of stored energy in animals [16]. Glycogen is the form of energy storage in animal body cells, and its synthesis and decomposition can maintain normal blood glucose levels. The liver and muscles of animals store the most glycogen, which are called liver glycogen and muscles glycogen respectively. After eating, excessive monosaccharides absorbed by the intestines are synthesized into glycogen and stored in the liver and muscles to avoid high blood glucose [30]. When the blood glucose concentration decreases after fasting, the liver glycogen is decomposed into glucose and enters the blood to supplement blood glucose [31]. The muscle glycogen is partially decomposed into lactic acid oxidation for energy supply, and most of the rest is circulated to the liver with blood, and is converted into liver glycogen through gluconeogenesis, thus supplementing blood glucose. Fasting for a long time before slaughter resulted in a large consumption of muscle glycogen. After slaughter, the glycogen content in muscles was less, and the lactic acid produced by glycolysis was also less. This study shows that the glycogen of the liver is almost exhausted after the fasting time is prolonged (≥6 h). With the extension of fasting time, the glycogen in the leg muscles showed a downward trend.

The blood glucose levels of broilers remains relatively stable, and the production and utilization are in a dynamic balance. After fasting, the absorption of glucose in the intestine decreases, the glycogen stored in the liver is decomposed quickly, the conversion of fat and protein is relatively slow, and the oxidation and energy supply of blood sugar increases, resulting in a sharp decrease in blood sugar levels [12]. This study showed that the prolongation of fasting time reduced the concentration of serum glucose.

After slaughter, the muscles oxygen supply is stopped, while the cell activity still needs energy under anaerobic conditions, which will lead to the accumulation of lactic acid. So, this study shows that the muscles pH value shows an upward trend with the prolongation of fasting time. The reason may be that after a long period of fasting, the glycogen consumption in the muscles supplies energy, the glycogen content in the muscles decreases, and the lactic acid produced by glycolysis decreases, resulting in a higher pH value [32]. The muscles pH value at 20 h after slaughter mainly depends on the muscles’ glycogen storage. This study shows that muscle glycogen is basically not consumed after fasting for 0 h, so muscles glycogen storage is more, lactic acid produced by glycolysis is also more, and muscle pH is lower at 20 h after slaughter. Prolonged fasting time (≥8 h) leads to glycogen metabolism and energy supply of muscles, resulting in higher pH value of muscles after slaughter. Some studies have shown that the L* value of muscles is significantly negatively correlated with pH [12,33]. In this experiment, the prolongation of fasting time before slaughter significantly increased the L* value of meat color and reduced the shear force, which is consistent with the research results of Partanen et al [34].

Serum albumin is the most abundant protein in plasma, which is synthesized in the liver and accounts for a large proportion of plasma protein. Its main function is to regulate blood osmotic pressure [35,36] The normal serum albumin levels of broilers remained relatively stable [37]. With the increase of fasting time, the absorption of protein in the intestine of broiler chickens decreases, and the rate of the liver albumin synthesis decreases [28]. This study shows that serum albumin concentration also decreases with fasting time. In this study, the activity of AST in broiler serum increased with the prolongation of fasting time, which may be due to the change of broiler metabolic activity after fasting, from anabolism to catabolism. ALT mainly exists in hepatocytes, and acute hepatocyte damage can cause a sharp increase in the activity of ALT enzyme in blood. This study shows that the activity of serum ALT decreases with the prolongation of fasting time, indicating that fasting for 24 hours does not cause damage to hepatocytes. Serum UA content is an index to evaluate the protein decomposition of birds [38]. In this study, the content of serum UA decreased with the prolongation of fasting time, which indicated that the oxidative decomposition of protein in fasted broilers decreased, which may be a protein retention mechanism.

## CONCLUSION

The results of this study show a significant influence of fasting time on carcass yield and meat quality in broilers. Moderate fasting (8 to 12 h) before slaughter can reduce the weight loss of broilers. Prolonged fasting (≥16 h) which increased body weight loss, decreased slaughtering performance and produced fluctuating blood indexes of broilers.

## Figures and Tables

**Table 1:** Effects of fasting time on carcass quality traits and body weight

Items	Treatments^1)^					SEM	p-value	
	
	F4	F8	F12	F16	F20		ANOVA	Linear
Initial body weight (g)	2,630.13	2,608.88	2,614.38	2,597.38	2,610.00	22.39	0.911	0.484
Slaughter body weight (g)	2,588.38^a^	2,538.63^ab^	2,525.38^ab^	2,495.50^b^	2,496.75^b^	22.70	0.063	0.004
Body weight loss (g)	41.75^e^	70.25^d^	89.00^c^	101.88^b^	113.25^a^	2.00	0.001	0.001
Weight loss rate (%)	1.59^e^	2.70^d^	3.41^c^	3.92^b^	4.34^a^	0.08	0.001	0.001
Carcass yield (%)	73.43^b^	74.80^ab^	75.57^a^	76.52^a^	76.14^a^	0.62	0.019	0.001
Breast muscles yield (%)	22.37^c^	22.34^c^	22.76^bc^	23.47^ab^	24.11^a^	0.33	0.005	0.001
Thigh yield (%)	29.71^b^	31.01^ab^	31.03^ab^	31.57^ab^	32.30^a^	0.58	0.076	0.005
Abdominal fat yield (%)	2.12	2.17	2.09	2.20	2.24	0.05	0.389	0.141

SEM, standard error of the mean; ANOVA, analysis of variance.

1) F4, transportation for 2 h, and laboratory rest for 2 h; F8, fasting for 4 h, transportation for 2 h, and laboratory rest for 2 h; F12, fasting for 8 h, transportation for 2 h, and laboratory rest for 2 h; F16, fasting for 12 h, transportation for 2 h, and laboratory rest for 2 h; F20, fasting for 16 h, transportation for 2 h, and laboratory rest for 2 h.

a–e Means with different letters within a row differ significantly (p<0.05).

**Table 2:** Effects of fasting time on meat quality traits of breast muscles

Items	Treatments^1)^					SEM	p-value	
	
	F4	F8	F12	F16	F20		ANOVA	Linear
L*	46.27^a^	45.61^ab^	45.38^bc^	45.20^bc^	44.57^c^	0.27	0.005	0.001
a*	2.72	2.70	2.72	2.71	2.66	0.04	0.887	0.433
b*	17.25	17.19	16.96	17.30	17.09	0.20	0.816	0.751
pH_45 min_	6.76^ab^	6.72^b^	6.80^ab^	6.81^a^	6.77^ab^	0.03	0.160	0.259
pH_24 h_	5.73^c^	5.78^bc^	5.88^ab^	5.89^ab^	5.96^a^	0.03	0.001	0.001
Drip loss (%)	2.89	3.00	2.94	3.00	3.02	0.06	0.651	0.246
Cooking loss (%)	32.76^a^	31.92^ab^	31.66^b^	31.62^b^	31.58^b^	0.28	0.043	0.007
Shear force (N)	13.59^a^	13.12^a^	11.99^b^	12.13^b^	12.01^b^	0.30	0.002	0.001
Moisture content (%)	75.31^a^	74.26^ab^	74.88^a^	73.66^ab^	72.60^b^	0.58	0.035	0.004

SEM, standard error of the mean; ANOVA, analysis of variance.

1) F4, transportation for 2 h, and laboratory rest for 2 h; F8, fasting for 4 h, transportation for 2 h, and laboratory rest for 2 h; F12, fasting for 8 h, transportation for 2 h, and laboratory rest for 2 h; F16, fasting for 12 h, transportation for 2 h, and laboratory rest for 2 h; F20, fasting for 16 h, transportation for 2 h, and laboratory rest for 2 h.

a–c Means with different letters within a row differ significantly (p<0.05).

**Table 3:** Effects of fasting time on meat quality traits of leg muscles

Items	Treatments^1)^					SEM	p-value	
	
	F4	F8	F12	F16	F20		ANOVA	Linear
L*	46.79^a^	46.34^ab^	45.68^b^	45.52^bc^	44.81^c^	0.26	0.001	0.001
a*	7.76	7.70	7.71	7.71	7.72	0.03	0.797	0.487
b*	11.88	11.95	11.92	12.05	11.71	0.22	0.889	0.751
pH_45 min_	6.53^b^	6.57^ab^	6.60^ab^	6.60^ab^	6.64^a^	0.03	0.101	0.007
pH_24 h_	6.07^b^	6.27^a^	6.28^a^	6.30^a^	6.33^a^	0.03	0.001	0.001
Drip loss (%)	2.51^b^	2.54^ab^	2.57^a^	2.57^a^	2.55^ab^	0.02	0.125	0.043
Cooking loss (%)	31.08	30.44	30.73	29.90	29.95	0.35	0.139	0.020
Shear force (N)	14.18	13.71	13.21	13.24	13.51	0.29	0.181	0.071
Moisture content (%)	76.09^a^	75.06^ab^	74.55^abc^	73.51^bc^	72.40^c^	0.69	0.013	0.001

SEM, standard error of the mean; ANOVA, analysis of variance.

1) F4, transportation for 2 h, and laboratory rest for 2 h; F8, fasting for 4 h, transportation for 2 h, and laboratory rest for 2 h; F12, fasting for 8 h, transportation for 2 h, and laboratory rest for 2 h; F16, fasting for 12 h, transportation for 2 h, and laboratory rest for 2 h; F20, fasting for 16 h, transportation for 2 h, and laboratory rest for 2 h.

a–c Means with different letters within a row differ significantly (p<0.05).

**Table 4:** Effect of pre-slaughter fasting time on glycometabolism of broilers

Items	Treatments^1)^					SEM	p-value	
	
	F4	F8	F12	F16	F20		ANOVA	Linear
Blood glucose (mg/L)	10.89	10.52	10.11	9.78	9.83	0.37	0.230	0.021
Hepatic glycogen (mg/g)	8.00^a^	2.20^b^	0.56^b^	0.51^b^	0.37^b^	0.79	0.001	0.001
Breast muscles glycogen (mg/g)	2.42	2.45	2.54	2.23	2.26	0.11	0.289	0.149
Leg muscles glycogen (mg/g)	0.99^a^	0.51^b^	0.37^b^	0.31^b^	0.34^b^	0.08	0.001	0.001

SEM, standard error of the mean; ANOVA, analysis of variance.

1) F4, transportation for 2 h, and laboratory rest for 2 h; F8, fasting for 4 h, transportation for 2 h, and laboratory rest for 2 h; F12, fasting for 8 h, transportation for 2 h, and laboratory rest for 2 h; F16, fasting for 12 h, transportation for 2 h, and laboratory rest for 2 h; F20, fasting for 16 h, transportation for 2 h, and laboratory rest for 2 h.

a,b Means with different letters within a row differ significantly (p<0.05).

**Table 5:** Effect of fasting time on serum biochemical indexes of broilers

Items	Treatments^1)^					SEM	p-value	
	
	F4	F8	F12	F16	F20		ANOVA	Linear
TP (g/L)	30.51	30.76	29.04	30.10	29.10	1.02	0.713	0.307
ALB	13.49	13.25	12.97	12.53	12.29	0.54	0.564	0.080
AST (U/L)	199.71^b^	217.89^b^	207.91^b^	254.35^a^	258.69^a^	7.38	0.001	0.001
ALT (U/L)	5.32^ab^	5.36^a^	5.19^ab^	5.02^ab^	4.65^b^	0.20	0.166	0.018
UA (μmol/L)	291.36^a^	272.62^ab^	275.04^ab^	242.09^b^	253.84^ab^	11.34	0.054	0.009
TC (mmol/L)	4.05	3.99	4.07	4.03	4.07	0.05	0.875	0.705
HDLC (mmol/L)	2.93	2.95	2.97	2.96	2.95	0.03	0.935	0.605
LDLC (mmol/L)	0.63	0.61	0.64	0.62	0.63	0.01	0.303	0.701
TG (mmol/L)	0.45^a^	0.40^ab^	0.38^ab^	0.34^bc^	0.29^c^	0.02	0.002	0.001

SEM, standard error of the mean; ANOVA, analysis of variance; TP, total protein; ALB, albumin; AST, aspartate transaminase; ALT, alanine aminotransferase; UA, uric acid; TC, total cholesterol; HDLC, high-density lipoprotein cholesterol; LDLC, low-density lipoprotein cholesterol cholesterol; TG, triglycerides.

1) F4, transportation for 2 h, and laboratory rest for 2 h; F8, fasting for 4 h, transportation for 2 h, and laboratory rest for 2 h; F12, fasting for 8 h, transportation for 2 h, and laboratory rest for 2 h; F16, fasting for 12 h, transportation for 2 h, and laboratory rest for 2 h; F20, fasting for 16 h, transportation for 2 h, and laboratory rest for 2 h.

a–c Means with different letters within a row differ significantly (p<0.05).
